# Neural network analysis of neutron and X-ray reflectivity data incorporating prior knowledge

**DOI:** 10.1107/S1600576724002115

**Published:** 2024-03-31

**Authors:** Valentin Munteanu, Vladimir Starostin, Alessandro Greco, Linus Pithan, Alexander Gerlach, Alexander Hinderhofer, Stefan Kowarik, Frank Schreiber

**Affiliations:** a University of Tübingen, Auf der Morgenstelle 10, 72076 Tübingen, Germany; b Deutsches Elektronen-Synchrotron DESY, Notkestraße 85, 22607 Hamburg, Germany; c Department of Physical Chemistry, University of Graz, Heinrichstraße 28, 8010 Graz, Austria; Montanuniversität Leoben, Austria

**Keywords:** reflectometry, machine learning, inverse problems, soft matter

## Abstract

Due to the ambiguity related to the lack of phase information, determining the physical parameters of multilayer thin films from measured neutron and X-ray reflectivity curves is, on a fundamental level, an underdetermined inverse problem. This ambiguity poses limitations on standard neural networks, constraining the range and number of considered parameters in previous machine learning solutions. To overcome this challenge, a novel training procedure has been designed which incorporates dynamic prior boundaries for each physical parameter as additional inputs to the neural network. In this manner, the neural network can be trained simultaneously on all well-posed subintervals of a larger parameter space in which the inverse problem is underdetermined. During inference, users can flexibly input their own prior knowledge about the physical system to constrain the neural network prediction to distinct target subintervals in the parameter space. The effectiveness of the method is demonstrated in various scenarios, including multilayer structures with a box model parameterization and a physics-inspired special parameterization of the scattering length density profile for a multilayer structure. In contrast to previous methods, this approach scales favourably when increasing the complexity of the inverse problem, working properly even for a five-layer multilayer model and a periodic multilayer model with up to 17 open parameters.

## Abstract 2

This study addresses the ambiguity related to the phase problem as a primary obstacle in machine-learning-based approaches for extracting information from X-ray reflectivity and neutron reflectivity data. The proposed solution is a procedure that enables training of a neural network on a continuous range of smaller subspaces of a large parameter space.

## 1. Introduction

X-ray and neutron reflectometry (XRR and NR, respectively) are well established and indispensable experimental techniques commonly used to investigate the scattering length density (SLD) profile along the direction perpendicular to the surface of samples such as thin films and multilayers (Tolan, 1999 ▸; Holý *et al.*, 1999 ▸; Sinha & Pynn, 2002 ▸; Daillant & Gibaud, 2009 ▸; Zhou & Chen, 1995 ▸; Benediktovich *et al.*, 2014 ▸). The most common way of modelling the SLD profile of a measured sample is via a box model parameterization, where the physical parameters of interest are the thickness, the roughness and the constant SLD of each layer in a multilayer structure. More complex parameterizations of the SLD profile can be employed, based on pre-existing physical knowledge or intuition about the investigated structure. For example, the interfaces in real layered systems can exhibit imperfections due to chemical diffusion or the formation of aggregates which cannot be modelled as roughness. XRR and NR have been extensively used in both *in situ* and *ex situ* studies of a large variety of systems, such as liquid and solid thin films (Kowarik *et al.*, 2006 ▸; Woll *et al.*, 2011 ▸; Braslau *et al.*, 1988 ▸; Metzger *et al.*, 1994 ▸; Michely & Krug, 2004 ▸; Lehmkühler *et al.*, 2008 ▸; Seeck *et al.*, 2002 ▸; Fragneto-Cusani, 2001 ▸; Festersen *et al.*, 2018 ▸; Schlomka *et al.*, 1996 ▸; Treece *et al.*, 2019 ▸), layers of polymers (Ankner *et al.*, 1993 ▸; Mukherjee *et al.*, 2002 ▸), lipids (Neville *et al.*, 2006 ▸; Skoda *et al.*, 2017 ▸; Salditt & Aeffner, 2016 ▸; Sironi *et al.*, 2016 ▸), self-assembled monolayers (Wasserman *et al.*, 1989 ▸; Skoda *et al.*, 2022 ▸; Chu *et al.*, 2020 ▸), and organic solar cells (Tidswell *et al.*, 1990 ▸; Fenter *et al.*, 1997 ▸; Lorch *et al.*, 2015 ▸). In addition, polarized NR (Majkrzak, 1991 ▸) can be used to study the magnetic properties of thin films. However, it is important to realize that these successful uses of reflectometry usually involve some form of complementary information in the data analysis, such as typical densities or reasonable intervals for the film thickness, provided by the experimentalist.

Reflectometry can be counted among the techniques affected by the phase problem. In the absence of complementary information, the lack of phase information introduces a degree of ambiguity when trying to reconstruct the SLD profile of the investigated sample from the measured reflectivity curve, *i.e.* the inverse problem is, on a fundamental level, underdetermined (‘ill-posed’). This intrinsic ambiguity of the scattering method is further magnified by additional ambiguity due to the limited experimental accuracy, such as instrument noise, measurement artefacts and the finite number of measured points over the domain of the momentum transfer *q*_*z*_ [*q* = (4π/λ)sinθ, where θ is half the scattering angle and λ is the wavelength of the incident radiation].

In recent years, machine learning has emerged as an alternative to classical methods of analysing surface scattering data (Hinderhofer *et al.*, 2023 ▸), being attractive due to its very fast prediction times and its ability to be incorporated into the operating pipelines of large-scale measurement facilities. In particular, fast machine-learning-based solutions are ideal for enabling an experimental feedback loop during reflectometry measurements to be performed in real time (Pithan *et al.*, 2023 ▸; Ritley *et al.*, 2001 ▸). While many machine learning approaches dedicated to reflectivity exist (Greco *et al.*, 2019 ▸, 2021 ▸, 2022 ▸; Mironov *et al.*, 2021 ▸; Doucet *et al.*, 2021 ▸; Aoki *et al.*, 2021 ▸; Kim & Lee, 2021 ▸; Andrejevic *et al.*, 2022 ▸), most of them do not directly address the inherent ambiguity of the reflectivity data, instead training neural networks over specific parameter domains where the ambiguity is not prominent enough to prevent convergence. Such networks lack the flexibility to be used in broader scenarios, typically requiring to be retrained on new parameter domains for each use case. Specifically, the work of Greco *et al.* (2022 ▸), while successful within its target, is limited to the case of a layer grown on top of a fixed substrate (silicon).

In our method proposed here, we enhance the solution of the inverse problem by including prior boundaries for the parameters as supplementary inputs to the neural network. This allows the network to be trained over wide parameter domains while the regression is conducted over small enough subdomains, defined by the prior bounds, to mitigate ambiguity. During inference, the output of the network is defined not only by the measured reflectivity curve but also by the prior experimental knowledge for the considered physical scenario. Different choices of prior bounds can lead to the recovery of distinct solution branches from the larger parameter domain.

The absence of extensive and diverse reflectometry data sets makes the use of experimental data for training purposes difficult. Consequently, simulations serve as a suitable alternative, a standard established in previous publications (Greco *et al.*, 2019 ▸; Mironov *et al.*, 2021 ▸; Doucet *et al*., 2021 ▸). The domain gap between experimental and simulated reflectivity curves can be mitigated by augmenting the simulations with physically inspired noise and distortions. We note that a combined training strategy such as online learning could potentially be used to adapt a trained model directly during beamtime to the unique noise characteristics inherent to the measurement instrument (Babu *et al.*, 2022 ▸).

Another limitation of existing approaches is that they require a specific discretization of the reflectivity curves, as imposed by common network architectures. While experimental curves can be interpolated to the required discretization, interpolation is prone to introducing unphysical artefacts, for example around the deep minima of Kiessig fringes in NR/XRR curves. To address this limitation, we introduce the use of a neural operator for processing reflectivity curves with variable discretizations (number of points and *q* ranges).

In this paper, we first discuss the theoretical concepts necessary to understand our approach in Section 2, after which we present the technical details of the implementation in Section 3. Finally, we demonstrate the results of our method for different parameterizations of the SLD profile (two-layer box model, five-layer box model, physics-informed model with *N* repeating monolayer units) on both simulated and experimental reflectivity curves in Section 4.

## 2. Theoretical considerations

### 2.1. The phase problem

This section provides a comprehensive description of the phase problem in reflectometry.

The phase problem is a ubiquitous issue for experimental techniques utilizing the interference of waves *A*exp(−*i*ω*t*) as a means of probing the physical properties of materials, caused by the fact that detectors cannot record the phase of the signal but only its intensity |*A*|^2^ (Volostnikov, 1990 ▸). This loss of information introduces a degree of ambiguity when trying to reconstruct the physical quantities of interest from the measured signal. It is well known that NR and XRR are among the techniques affected by the phase problem (Kozhevnikov, 2003 ▸), which precludes an analytical solution via the Gel’fand–Levitan–Marchenko (Newton, 1974 ▸) inverse scattering equation. Some approaches for experimentally tackling the phase problem of NR and XRR have been developed, such as the reference layer method (Majkrzak *et al.*, 1998 ▸) and the Lloyd mirage technique (Allman *et al.*, 1994 ▸), but they have certain practical limitations. Of particular interest is the use of magnetic reference layers (Masoudi & Pazirandeh, 2005 ▸) in polarized neutron reflectometry where the SLD of the reference layer depends on the polarization of the incident neutrons. Three distinct measurements for up, down and non-polarized neutron beams then allow for the determination of the phase. Also for NR, the amount of structural detail extracted from reflectivity data can be maximized by flexibly varying the SLD of specific structures within the sample via isomorphic isotopic substitution (Heinrich, 2016 ▸), such as the replacement of protium with deuterium (selective deuteration) in the hydrocarbon chains of lipid bilayers (Clifton *et al.*, 2012 ▸). Alternatively, the degree of ambiguity can be reduced when measuring a series of reflectivity curves for a sample with evolving structure (*e.g.* during film deposition). Such theoretical ambiguity is in practice further accentuated by experimental sources of error (noise, finite instrumental resolution, artefacts) and the discrete nature of the measurement process, the scattered intensity being recorded over a finite range of the momentum transfer *q*_*z*_ at a finite number of points.

A consequence of this ambiguity is the underdetermined (‘ill-posed’) nature of parameter recovery from the measured data, different SLD profiles corresponding to equivalent reflectivity curves. When taking into account multiple scattering at the interfaces, as described by Parratt’s recursive formalism (Parratt, 1954 ▸) or the Abelès transfer-matrix method (Abelès, 1950 ▸), some information about the phase can be recovered in the low-*q*_*z*_ region (Zhou & Chen, 1993 ▸). However, the difference between reflectivity curves can still become vanishingly small (Pershan, 1994 ▸), especially when compounded with systematic measurement errors of the total reflection edge.

In the following, we briefly summarize a theoretical derivation of how such SLD profiles can be identified in the mathematically simpler kinematic approximation, which neglects multiple scattering at the sample interfaces so that the calculation remains analytical. In the kinematic approximation, the scattered intensity is proportional to the square of the total scattering amplitude: 

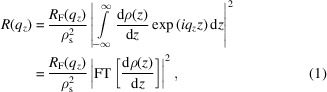

where *R*_F_(*q*_*z*_) denotes the Fresnel reflectivity, ρ(*z*) is the SLD profile along the perpendicular direction *z* to the sample and ρ_s_ is the SLD of the substrate.

For the commonly used box model with interfacial roughness parametrized via the Névot–Croce factor (Névot & Croce, 1980 ▸), an SLD profile can be written as a sum of the error functions:

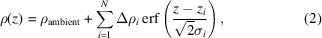

where erf(*z*) is the error function, *N* the number of interfaces, *z*_*i*_ the position of the *i*th interface, σ_*i*_ the roughness of the *i*th interface, and Δρ_*i*_ the SLD difference between interfaces *i* and *i* + 1.

By substituting equation (2) into equation (1), we can explicitly calculate the Fourier transform:

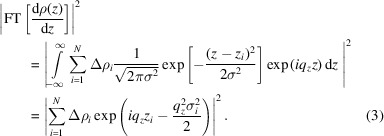




Finally, we obtain a decomposition of the scattered intensity into the sum of a constant term and several sinusoidal components with amplitudes 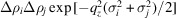 and frequencies Δ*z*_*ij*_ = *z*_*i*_ − *z*_*j*_:

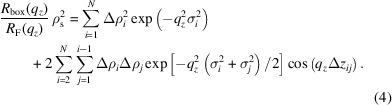




**Figure 1:**
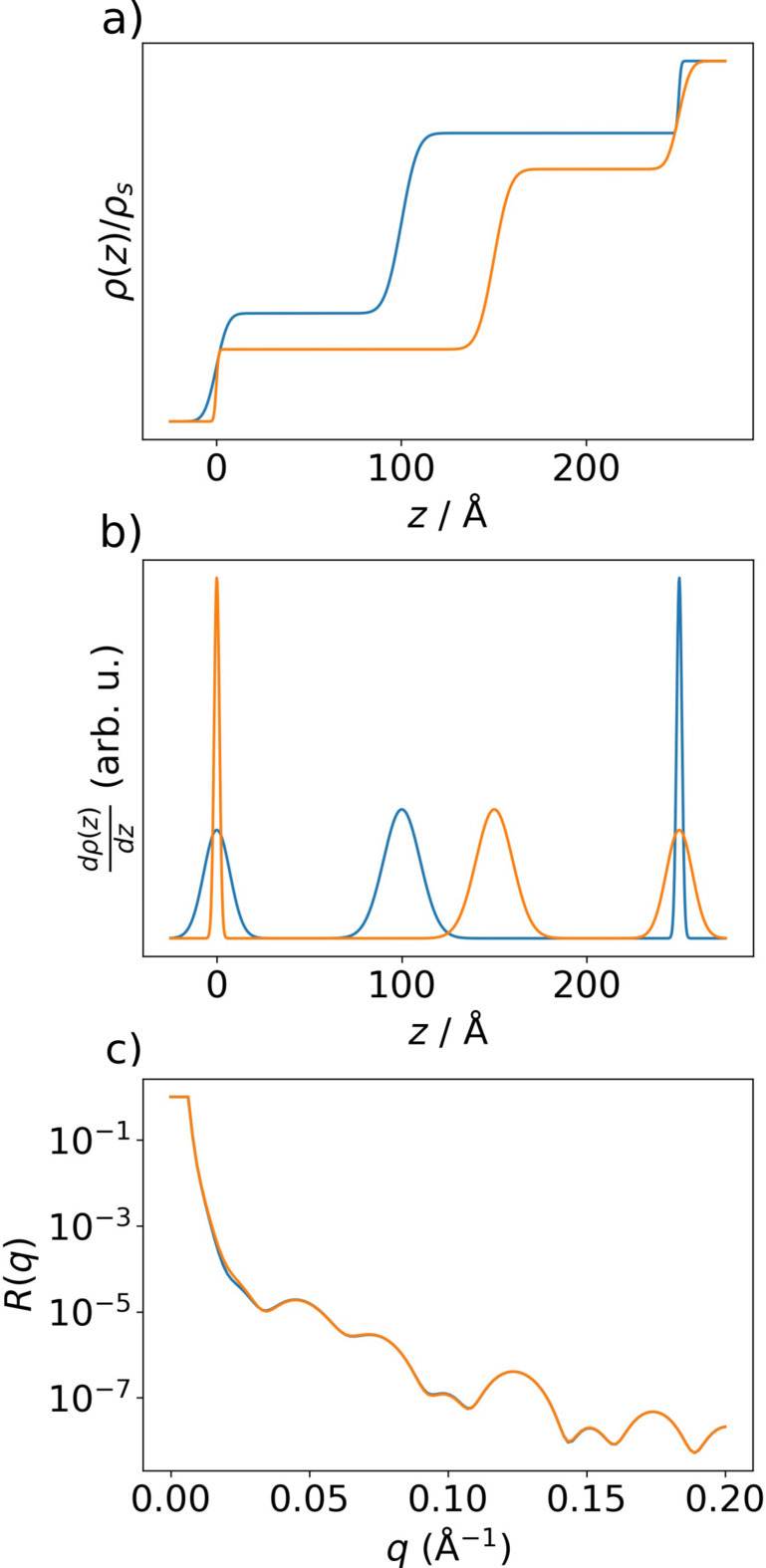
(*a*) Two SLD profiles with mirrored derivatives. (*b*) The derivatives of the two SLD profiles. (*c*) The corresponding reflectivity curves for the two SLD profiles are almost identical (they would be exactly identical in the kinematic approximation), despite the fact that the SLD profiles in (*a*) are very dissimilar.

By solving for all combinations of Δρ_*i*_ and *z*_*i*_ with the same scattered intensity, one can identify classes of theoretical identical solutions in the kinematic approximation, such as profiles with mirrored derivatives as reported in earlier studies (Pershan, 1994 ▸; Sivia *et al.*, 1991 ▸; Pynn, 1992 ▸) (shown in Fig. 1 ▸). Restricting the shape of the considered SLD profiles to a box model with a fixed number of layers already imposes a strong constraint on the space of admissible solutions.

### 2.2. Solving ill-posed inverse problems using neural networks

If a forward process *f*(**x**) = **y** maps the hidden parameters **x** describing a physical system to an observable signal **y**, the inverse problem can be described as retrieving the physical parameters from a possibly noise-corrupted measurement of the signal (Kabanikhin, 2008 ▸). When the inverse problem *f* ^−1^(**y**) = **x** represents a one-to-one mapping, neural networks can be straightforwardly trained to approximate the inverse function. In contrast, a many-to-one mapping of the forward process leads to an underdetermined ill-posed inverse problem, and the corresponding one-to-many inverse mapping *f* ^−1^(**y**) cannot be approximated via regression. Attempting to train a neural network as a point estimator over the domain containing the non-uniqueness would lead to incorrect predictions corresponding to an average of the distinct solution branches. Different machine learning approaches exist for addressing ill-posed inverse problems (Adler & Öktem, 2017 ▸; Li, Schwab *et al.*, 2020 ▸; Ardizzone *et al.*, 2019 ▸) but they generally depend on the specific application.

The present study proposes a novel approach for tackling ill-posed inverse problems which takes advantage of the *a priori* knowledge of the experimentalist. The conventional methodology for analysing reflectometry data, as implemented in commonly used software such as *GenX* (Glavic & Björck, 2022 ▸) or *refnx* (Nelson & Prescott, 2019 ▸), involves setting prior boundaries for the parameters describing the SLD profile of the sample and running an optimization algorithm, such as differential evolution, resulting in a single solution for the values of the parameters. Our machine learning approach draws inspiration from this classical procedure and combines the high speed of neural networks with the flexibility of conventional fitting procedures. Our proposed method is applicable to cases where enough prior knowledge about the sample is available such that a single mode of the distribution is isolated and the other solution branches can be discarded.

Typically, some physical parameters of the studied samples are known to the experimentalist with narrow uncertainty ranges, such as densities of the used materials, thicknesses of the deposited layers *etc*. In conventional fitting procedures, this information is used to set upper and lower bounds on each sample parameter within which solutions are allowed, the bounds being narrower for layer SLDs and wider for layer thicknesses and roughnesses. Our implementation allows us to provide such prior knowledge as input to the neural network in the form of two prior bounds for each parameter; the regression problem is constrained to the local domain defined by the prior bounds, thus avoiding the non-uniqueness associated with the full parameter domain. As long as the prior bounds are narrow enough not to include multiple solution branches, the inverse mapping is well determined and can be approximated by a neural network. In practice, even a small amount of prior knowledge can be enough to rule out ambiguity in reflectometry analysis. We note that narrow priors do not guarantee a single solution, as multiple solutions might occur close to each other. However, our general assumption, shared by experimentalists who analyse reflectometry data via conventional fitting, is that for a large set of cases the prior information is sufficient to isolate a single solution.

Our method can be envisioned as simultaneously learning the inverse problem on all possible subdomains (or a subset of subdomains) of the full parameter domain, using a single neural network. Thus, some parallels can be drawn with approaches such as that of Bae *et al.* (2022 ▸) where a beta variational autoencoder is trained simultaneously for all values of the β coefficient using a single neural network (a task which would typically require retraining for each β value).

Importantly, any prediction provided by a neural network should be validated for physical consistency, taking into account the specifics of the investigated samples. Furthermore, the initial neural network prediction can be further refined using conventional fitting techniques or provided as a starting point for posterior sampling using Markov-chain Monte Carlo techniques (Gelman *et al.*, 2013 ▸).

### 2.3. Discretization-invariant learning

Neural networks can only learn mappings between finite-dimensional vector spaces, some architectures such as the multilayer perceptron requiring a fixed discretization (range and resolution) of the input. A new paradigm is represented by neural operators, which can learn mappings between infinite-dimensional function spaces, allowing discretization-invariant learning (Li, Kovachki *et al.*, 2020 ▸; Kovachki *et al.*, 2023 ▸). For an input function *v*_0_(*x*) defined over a domain *D*, a neural operator is constructed as a series of transformations 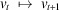 , 

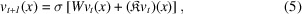

where *W* is a linear transformation, σ is a nonlinear activation function and 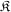 is a non-local integral operator with learnable kernel 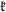 :

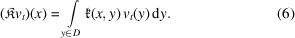




The Fourier neural operator (FNO) (Li *et al.*, 2021 ▸) represents an efficient and expressive implementation of a neural operator which imposes 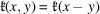 and makes use of the convolution theorem. It parameterizes the kernel operator directly in Fourier space as 

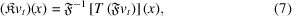

where 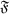 and 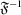 represent, respectively, the direct and the inverse discrete Fourier transform, and *T* is a learnable linear transformation.

Neural operators have been predominantly used in the fields of differential equation solving and physics-informed learning (Oommen *et al.*, 2022 ▸; Wen *et al.*, 2022 ▸). Here, we use a neural operator to learn a vector embedding for reflectivity curves with variable discretizations (*q* ranges and numbers of points) for our regression inverse problem. Such an approach is beneficial as it confers a higher degree of flexibility on the trained model, enabling the use of the full measured signal without relying on interpolation.

## 3. Design and implementation of our method

### 3.1. Training methodology and neural network architecture

To enable the solution of the inverse problem by confining the regression within a sample-dependent local domain, we generate ground-truth values of the parameters for training in the following manner. Firstly, for each parameter, we obtain the centre (*c*) and the width (*w*) of the local domain, the centre being uniformly sampled from the global domain (*i.e.* the parameter range) and the width being uniformly sampled from a predefined width range for that parameter. Secondly, the ground-truth values of the parameters are obtained by uniform sampling within the local domain [*c* − *w*/2, *c* + *w*/2] defined by the previously sampled values, the two prior bounds being the edges of this local domain, *c* − *w*/2 and *c* + *w*/2.

The whole training process is performed exclusively using simulated data. At each training step, we simulate a new batch of reflectivity curves from the sampled ground-truth parameters using a fast GPU-accelerated *Pytorch* (Paszke *et al.*, 2019 ▸) implementation of the Abelès transfer-matrix method. The training is conducted in a one-epoch regime (Komatsuzaki, 2019 ▸), the data not being reused at any step during the training. Consequently, the effective data set size is the product of the number of iterations and the batch size. As a preprocessing step, we set intensities below 10^−10^ which cannot be recorded in most experimental scenarios (although there are exceptions) to this chosen minimum threshold, we add noise to the curve, and we apply a logarithmic transformation followed by a linear rescaling. The prior bounds are normalized with respect to the corresponding parameter ranges and the ground-truth parameters are normalized with respect to the local domain they were sampled from, such that all the inputs and outputs to the neural network are in the range [−1, 1].

**Figure 2:**
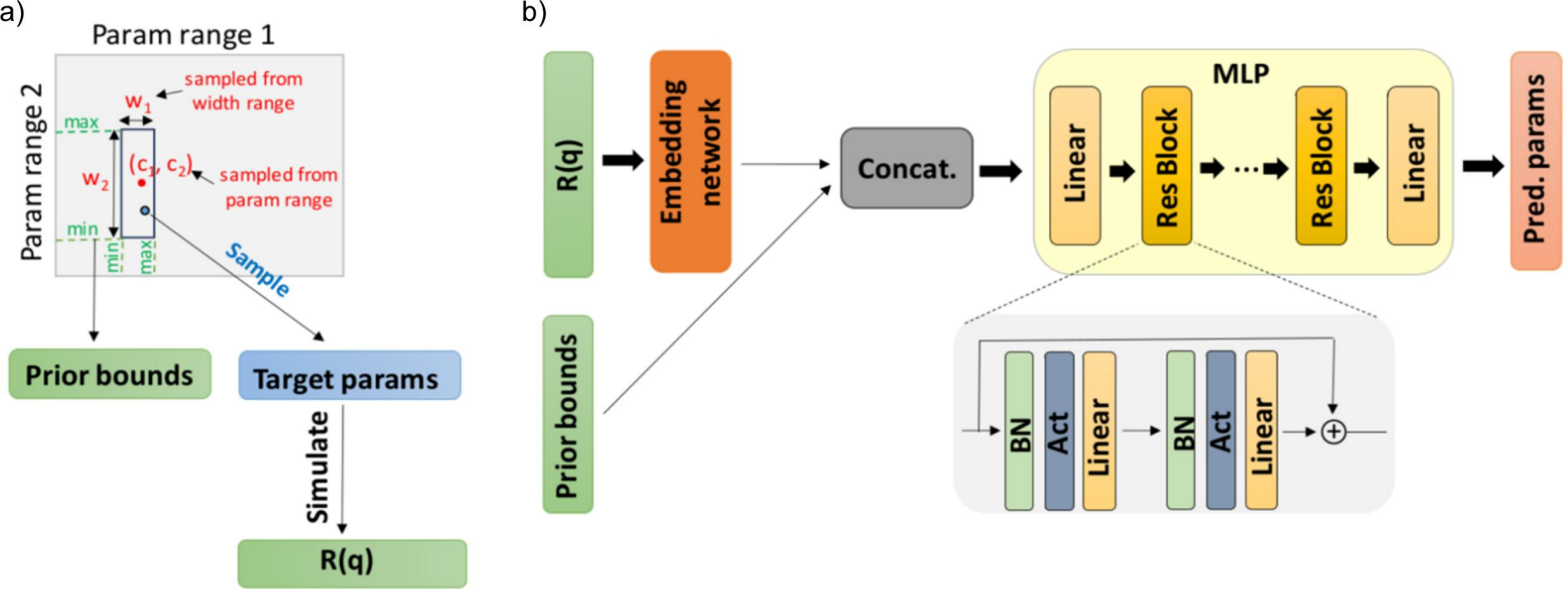
(*a*) Data generation for training. The prior bounds and ground-truth parameters are sampled using the described procedure and the reflectivity curves are simulated from the ground-truth parameters. (*b*) The neural network architecture. An embedding of the reflectivity curves and the prior bounds are provided as inputs to the MLP. The MLP consists of residual blocks with batch normalization (BN), nonlinear activation (chosen to be GELU) and linear layers.

As shown in Fig. 2 ▸, an embedding of the reflectivity curve and the prior bounds are input to a fully connected neural network (also known as a multilayer perceptron or MLP; Murtagh, 1991 ▸), the loss being computed as the mean-squared error between the neural network output and the ground-truth parameters (normalized with respect to the prior bounds). Note that obtaining the final prediction requires reversing the normalization of the neural network output with respect to the prior bounds. The architecture of our model and the sampling procedure are shown in Fig. 2 ▸ (parameter scaling omitted for simplicity). The MLP consists of a sequence of *n*_blocks_ = 6 residual blocks inspired by the ResNet (He *et al.*, 2016 ▸) architecture design, each block containing two hidden layers of width dim_hidden_ = 1024 neurons. The use of skip connections has the role of facilitating gradient propagation and preventing singularities (Orhan & Pitkow, 2018 ▸). Batch normalization (Ioffe & Szegedy, 2015 ▸) is known to improve the convergence of neural networks, so we use it to normalize the intermediate features before activation. Since the type of activation function can have a significant impact on the performance of a neural network, we explored the use of several popular activation functions [ReLU (Fukushima, 1975 ▸), GELU (Hendrycks & Gimpel, 2020 ▸), SELU (Klambauer *et al.*, 2017 ▸) and Mish (Misra, 2020 ▸)], choosing GELU as the default.

We trained our models using the AdamW (Loshchilov & Hutter, 2019 ▸) optimizer, a version of Adam (Kingma & Ba, 2017 ▸) with decoupled weight decay regularization. The weight decay coefficient is kept at the default value of 0.01. The initial learning rate of 0.0001 was decreased by a factor of 10 on plateau of the loss. We used the largest batch size that fits in our GPU memory (4096) to ensure stable gradients.

### 3.2. Embedding networks

Going beyond previous publications, where a reflectivity curve is directly provided as input to the MLP, we first use a network that produces a latent embedding of the reflectivity curve which is subsequently fed to the MLP. Our implementation is modular, allowing seamless replacement of the components of the model. A one-dimensional convolutional neural network (1D CNN) (Kiranyaz *et al.*, 2021 ▸) is the default embedding network used for our model. The FNO embedding network is provided as an alternative to the 1D CNN, allowing training for reflectivity curves with variable discretization, but its convergence is slower than that of the 1D CNN.

**Figure 3:**
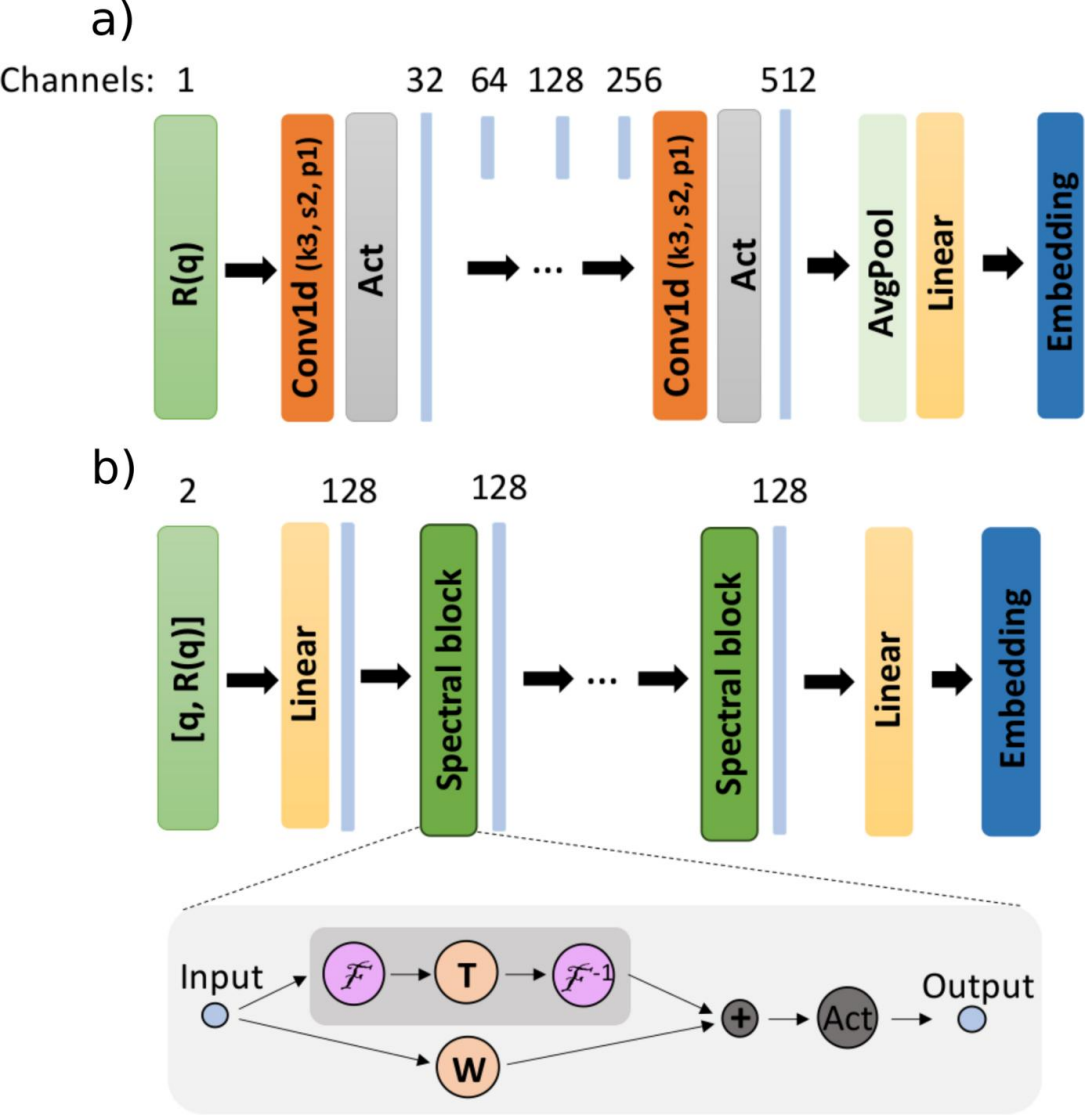
The architecture of the embedding networks. (*a*) The 1D CNN, consisting of convolutions with kernel size 3, stride 2 and padding 1. (*b*) The FNO, consisting of spectral blocks which implement the neural operator kernel in Fourier space.

When training a model on reflectivity curves with fixed discretization, a 1D CNN embedding network is parameter-efficient and makes better use of the sequential characteristics of the data. While less popular than their 2D counterparts, 1D CNNs have been successfully used in a variety of tasks involving 1D signals, such as automatic speech recognition (Collobert *et al.*, 2016 ▸) and time-series prediction (Guessoum *et al.*, 2022 ▸). The 1D CNN, as shown in Fig. 3 ▸(*a*), consists of a sequence of convolutional layers with kernel size 3, stride 2 and padding 1, the dimension of the signal being (approximately) halved after each layer. At the same time, the number of channels is doubled after each layer, starting from 32 up to ch_out_ = 512. An adaptive average pooling layer with output size dim_avpool_ = 8 ensures a fixed input size (ch_out_ × dim_avpool_) for a linear layer, which produces the final embedding with dimension dim_emb,CNN_ = 128. While the adaptive average pooling allows a fixed size embedding to be obtained from curves with variable discretizations, CNNs do not enable discretization-invariant learning, as shown in previous studies (Li *et al.*, 2021 ▸).

When training a model on reflectivity curves with variable discretizations, we employ an FNO as the embedding network [Fig. 3 ▸(*b*)], as theoretically motivated in the previous section on discretization-invariant learning. In this scenario the minimum and maximum values of *q* and the number of points in the curve are also uniformly sampled for each batch from the considered ranges. The input to the FNO is the reflectivity curve together with the corresponding *q* values (concatenated along the channel axis). After the input is raised to a higher channel space ch_FNO_ = 128 by a pointwise linear operation, a sequence of *n*_FNO_ = 5 spectral blocks are applied which implement the kernel operator in Fourier space as illustrated in Fig. 3 ▸(*b*). The number of Fourier modes kept after performing the discrete Fourier transform is a hyperparameter set at *n*_modes_ = 16. Finally, a mean pooling over the input dimension followed by a linear layer produces the last embedding with dimension dim_emb,FNO_ = 256.

## 4. Results

We trained neural networks according to the previously described approach for different parameterizations of a thin-film SLD profile. Each of the following subsections elaborates on a network trained on data with a different parameterization: either a different number of layers (two or five) for the box model or a physics-informed special parameterization of a multilayer structure with repeating monolayers. We use the 1D CNN as the default embedding network. In the last subsection the FNO is used as the embedding network for data with variable discretization. We evaluate performance metrics on statistically significant batches of simulated data and we illustrate the applicability of our method on experimental reflectivity data when available.

### 4.1. Two-layer box model

This subsection shows the performance evaluation for a neural network trained on data corresponding to a two-layer parameterization of the box model, the total number of physical parameters predicted by the neural network being eight [three parameters per layer (thickness, roughness, SLD) plus two additional parameters for the substrate (roughness and SLD)]. The *q* range of the simulated curves is [0.02, 0.15] Å^−1^ which aligns with the range of the test experimental data, and the resolution is 128 points. Various types of noise were applied to the simulated reflectivity curves in order to provide robustness to experimental artefacts, namely Poisson noise, *q*-position noise, curve shifting and curve scaling based on previous investigations (Greco *et al.*, 2021 ▸).

**Figure 4:**
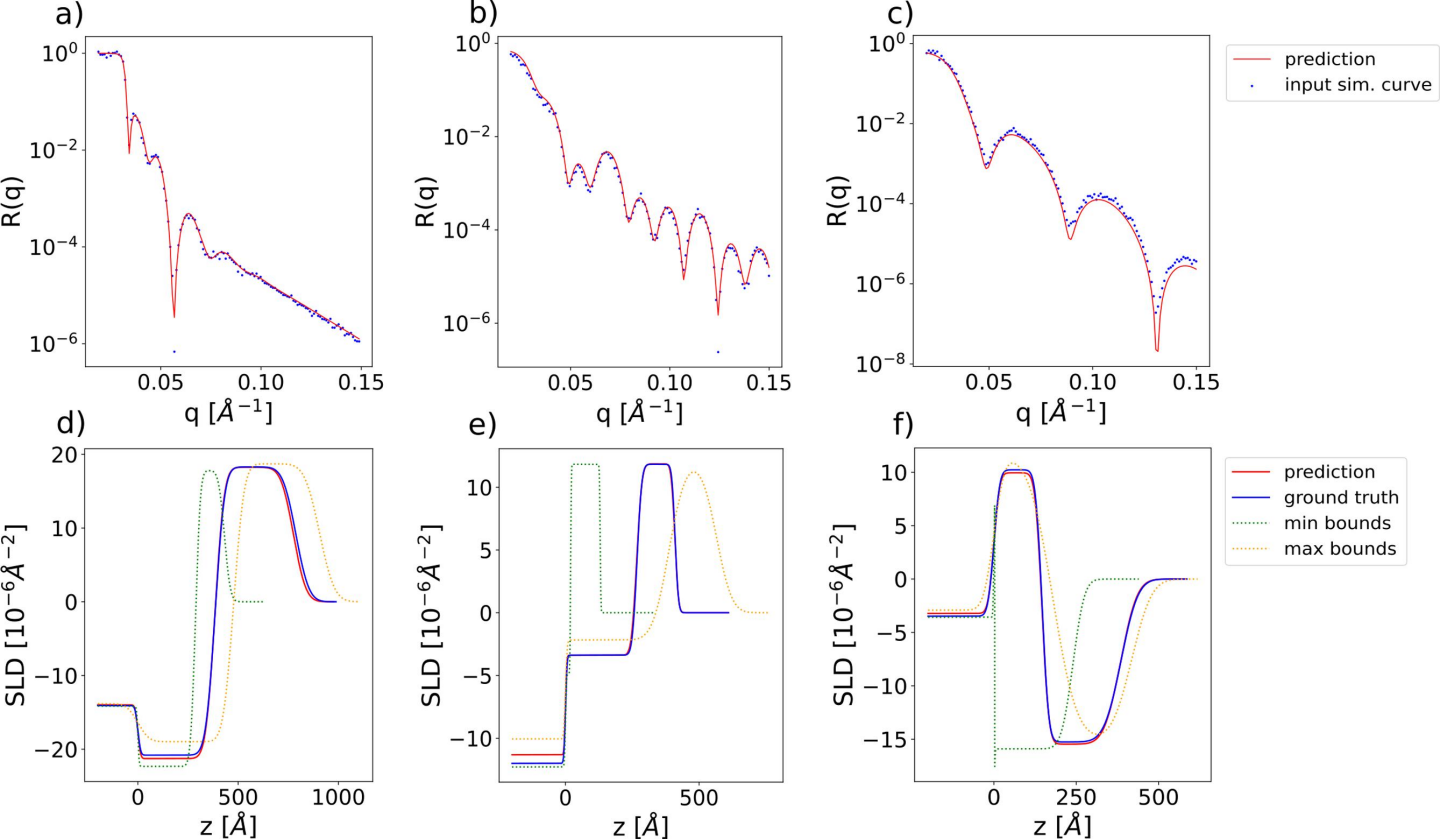
(*a*)–(*c*) Examples of input simulated reflectivity curves (blue markers) and the corresponding neural network predictions (red lines) for the two-layer model. (*d*)–(*f*) Ground-truth (blue) and predicted (red) SLD profiles corresponding to the reflectivity curves in the top row. SLD profiles corresponding to the minimum (green) and maximum (yellow) prior bounds used for the prediction are also shown as dotted lines. These minimum and maximum bound profiles should not be visually interpreted as an envelope encasing the ground-truth or predicted SLD profiles.

**Figure 5:**
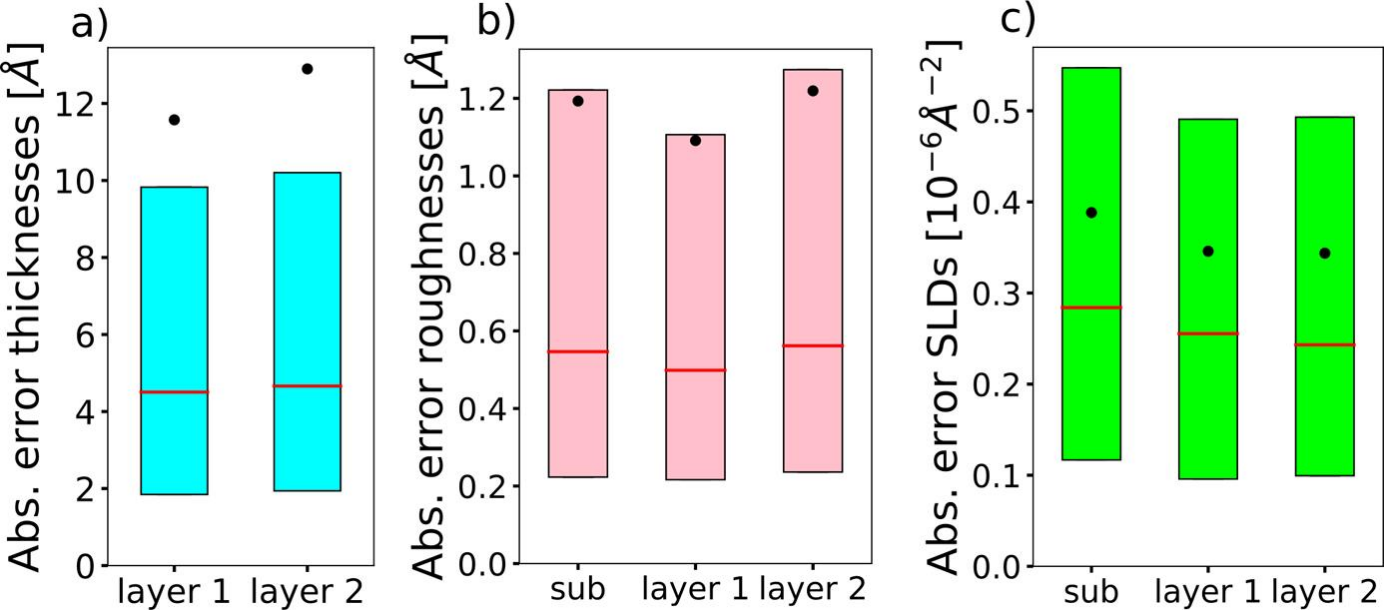
Box plots of the absolute errors of each predicted parameter of the two-layer model, computed over a batch of 4096 simulated curves, with the prior bounds being uniformly sampled. (*a*) Thicknesses, (*b*) roughnesses and (*c*) SLDs. The horizontal red lines denote the median, the black dots denote the mean, and the lower and upper extremities of the box plots denote the 25th and 75th percentiles, respectively.

The chosen parameter ranges are [0, 500] Å for the thicknesses, [0, 60] Å for the roughnesses and [−25, 25] × 10^−6^ Å^−2^ for the SLDs. The negative values of the SLD include specific cases of NR. The ranges of the prior bound widths are [0.01, 500] Å for the thicknesses, [0.01, 60] Å for the roughnesses and [0.01, 4] × 10^−6^ Å^−2^ for the SLDs. While the prior bound widths for the thicknesses and roughnesses span the whole domain of these parameter types, the maximum prior bound width for the SLDs was reduced, since this type of parameter is associated with the highest amount of prior experimental knowledge. Fig. 4 ▸ illustrates examples of input simulated reflectivity curves for this model, together with the neural network predictions (top row) and the ground-truth and predicted SLD profiles (bottom row). The minimum and maximum bound profiles serve as visual indicators of the target interval’s narrowness, as defined by the input prior bounds for each shown example. The minimum bound profile is obtained by setting the values of all parameters (thicknesses, roughnesses and layer SLDs) to their respective minimum prior values, and conversely for the maximum bound profile. Fig. 5 ▸ shows box plots of the absolute errors for each of the eight predicted parameters [panel (*a*) thicknesses, (*b*) roughnesses and (*c*) SLDs] computed over a batch of 4096 simulated curves, the ground-truth parameters and prior bounds being generated as in the training procedure. We can see from the box plots that the prediction errors for the thicknesses, roughnesses and SLDs are quite low considering the large parameter ranges used for training. We also note that the mean of the absolute errors for the thickness predictions is higher than both the median and the 75th percentile, which indicates the existence of several outlier predictions with a relatively high error.

**Figure 6:**
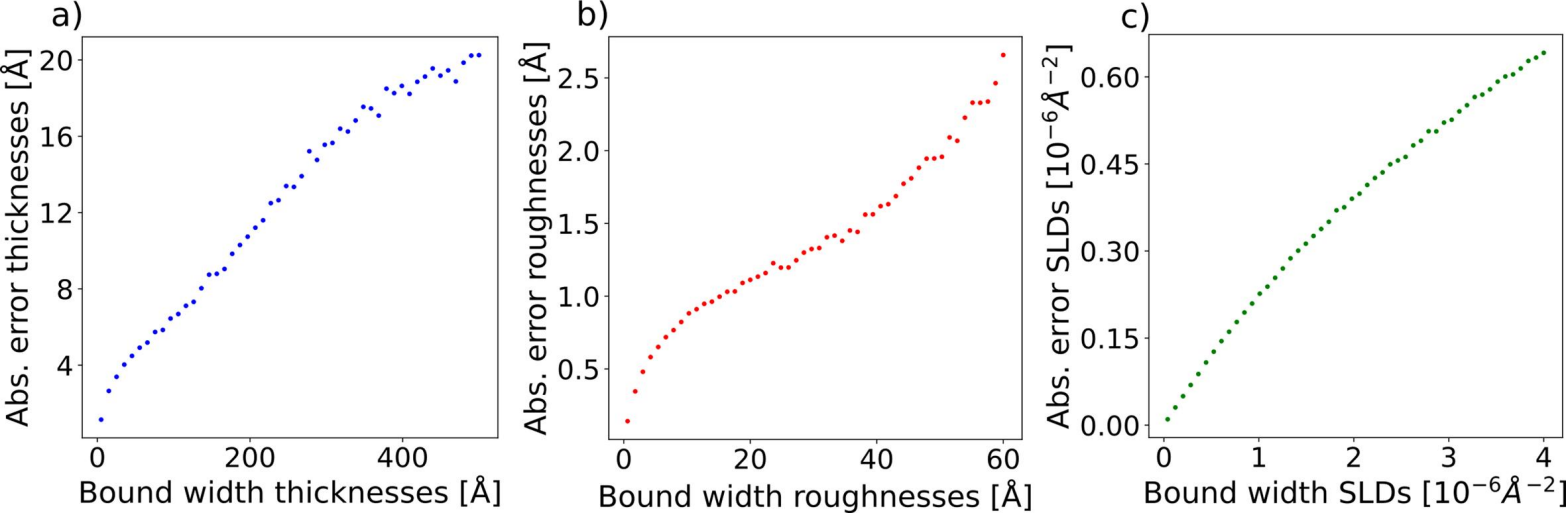
The dependence of the mean absolute error of each parameter type, (*a*) thickness, (*b*) roughness and (*c*) SLD, for the two-layer model as a function of the prior bound width.

It is important to understand how the performance of the model depends on the input prior bounds, as this informs users how much prior knowledge they should provide for an expected prediction quality. To evaluate such a dependence, we sample batches of ground-truth parameters and prior bounds such that the prior bound widths are fixed for each batch. The prior bound widths are varied by multiplying the maximum bound width of one parameter type at a time (while keeping the maximum bound width for the other parameter types constant) by a scalar value in the range [0, 1]. As shown in Fig. 6 ▸, we observe that the absolute errors of the thicknesses, roughnesses and SLDs decrease when the relative prior bound width for the corresponding parameter type is decreased, as expected. A relative bound width of 0 represents the trivial case where the prior bounds define the ground-truth parameters exactly.

**Figure 7:**
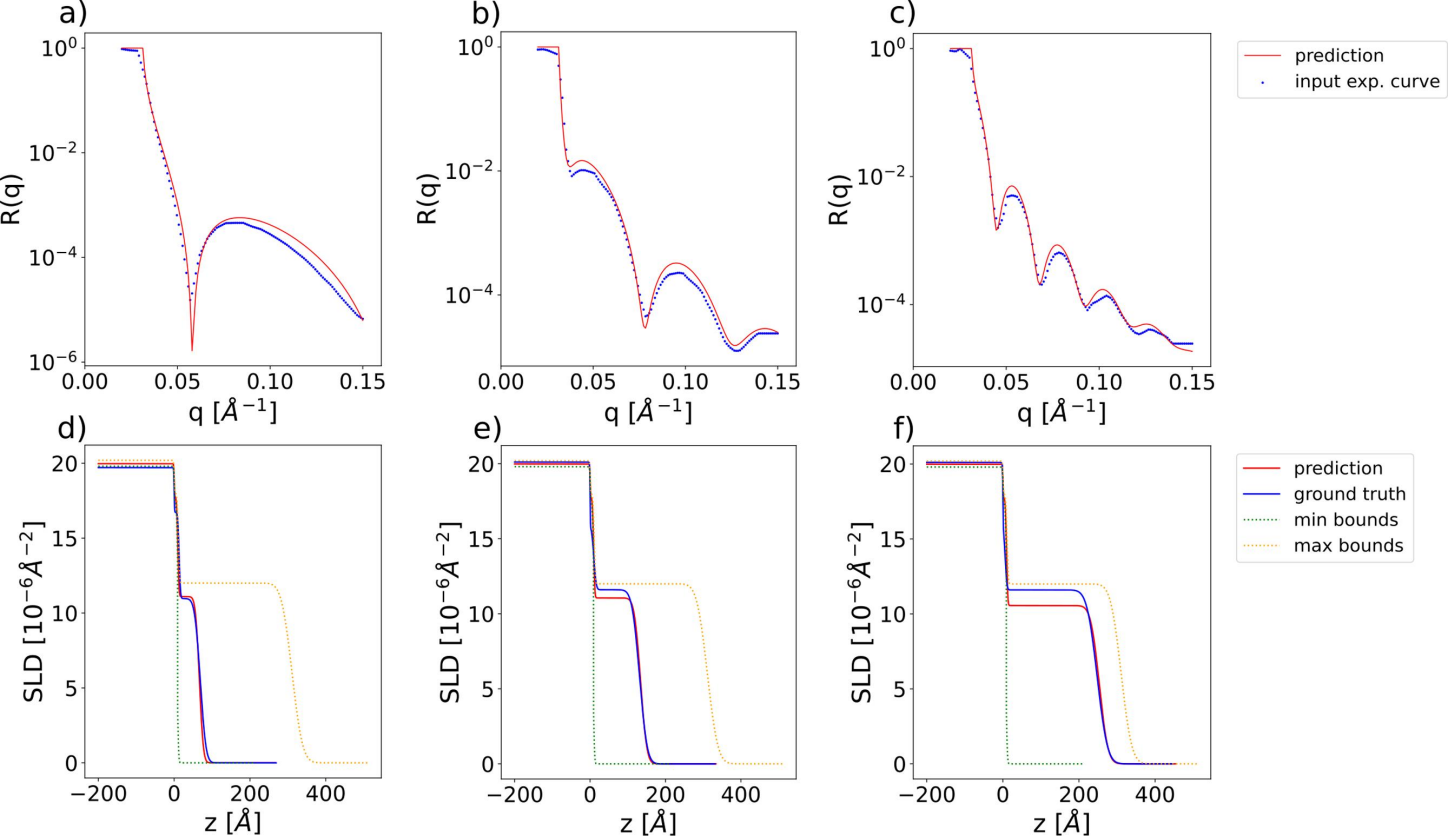
(*a*)–(*c*) Examples of input experimental reflectivity curves (blue markers) and the corresponding neural network predictions (red lines). (*d*)–(*f*) Ground-truth (blue) and predicted (red) SLD profiles corresponding to the reflectivity curves in the top row. SLD profiles corresponding to the minimum (green) and maximum (yellow) prior bounds used for the prediction are also shown as dotted lines. These minimum and maximum bound profiles should not be visually interpreted as an envelope encasing the ground-truth or predicted SLD profiles.

We demonstrate the applicability of our method for analysing experimental reflectivity data using XRR curves from a previously published data set (Pithan *et al.*, 2022 ▸) containing *in situ* measurements performed during the deposition of a layer of organic material [diindenoperylene (DIP) or pentacene] on top of a thin silicon oxide layer sitting on a silicon substrate, together with the ground-truth manual fits of the parameters. We are able to exploit the *a priori* experimental knowledge of this data set by setting narrow prior bounds around the known values of the SLD for the substrate (Si) and the bottom layer (SiO_2_). In previous approaches using this data set (Greco *et al.*, 2022 ▸), all the parameters of the substrate and SiO_2_ layer were kept at predefined constant values during training and only the thickness, roughness and SLD of the top layer were predicted. Our approach is capable of also tackling this specific use case while being applicable to broader scenarios, such as for substrates other than silicon. For the predictions on the experimental data we use the same trained network we previously employed for the evaluation on the simulated curves. Fig. 7 ▸ shows input experimental curves together with the neural network predictions (top row) and the corresponding SLD profiles (bottom row).

### 4.2. Five-layer box model

**Figure 8:**
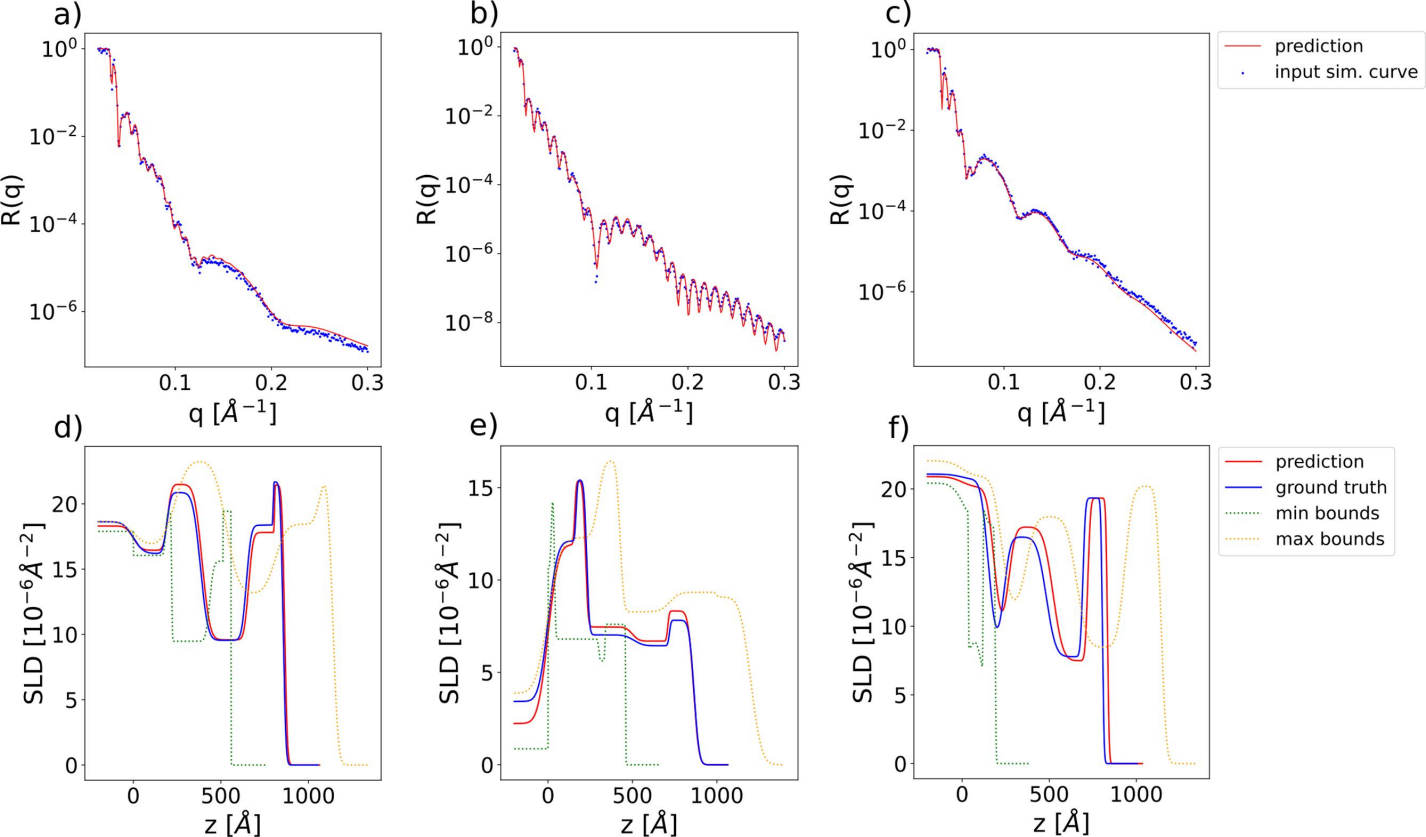
(*a*)–(*c*) Examples of input simulated reflectivity curves (blue markers) and the corresponding neural network predictions (red lines) for the five-layer model. (*d*)–(*f*) Ground-truth (blue) and predicted (red) SLD profiles corresponding to the reflectivity curves in the top row. SLD profiles corresponding to the minimum (green) and maximum (yellow) prior bounds used for the prediction are also shown as dotted lines. These minimum and maximum bound profiles should not be visually interpreted as an envelope encasing the ground-truth or predicted SLD profiles.

**Figure 9:**
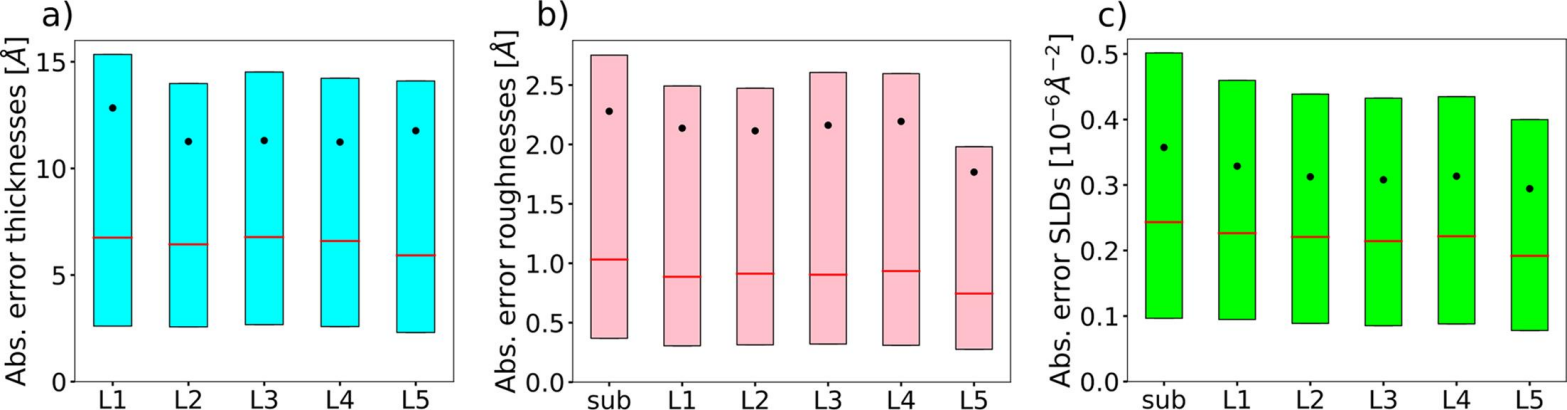
Box plots of the absolute errors of each predicted parameter of the five-layer model, computed over a batch of 4096 simulated curves, with the prior bounds being uniformly sampled. The horizontal red lines denote the median, the black dots denote the mean, and the lower and upper extremities of the box plots denote the 25th and 75th percentiles, respectively.

Increasing the number of layers in the box model parameterization increases the difficulty of training a neural network for the inverse problem since the reflectivity curves become more complex and the degree of non-uniqueness also increases. Nevertheless, we demonstrate that our method can still be successfully applied to models with an increased number of layers, namely a five-layer model, the total number of physical parameters predicted by the neural network being 17 [three parameters per layer (thickness, roughness, SLD) plus two additional parameters for the substrate (roughness and SLD)]. We increase the *q* range of the simulated curves to [0.02, 0.3] Å^−1^ and the resolution to 256 points. The chosen parameter ranges are [0, 300] Å for the thicknesses, [0, 60] Å for the roughnesses and [0, 25] × 10^−6^ Å^−2^ for the SLDs. The ranges of the prior bound widths are [0.01, 300] Å for the thicknesses, [0.01, 60] Å for the roughnesses and [0.01, 4] × 10^−6^ Å^−2^ for the SLDs. Fig. 8 ▸ shows examples of input simulated curves and predictions for the five-layer model, together with the corresponding SLD profiles. The absolute errors between the ground-truth and predicted parameters are displayed in Fig. 9 ▸. We observe that the prediction errors do not vary much with the specific layer in the model. While, as expected, the prediction errors for the five-layer model are higher than those for the two-layer model (the difference being more pronounced for roughnesses and less pronounced for SLDs), the performance of our model is still very good in this more challenging use case.

### 4.3. Complex multilayer model

**Figure 10:**
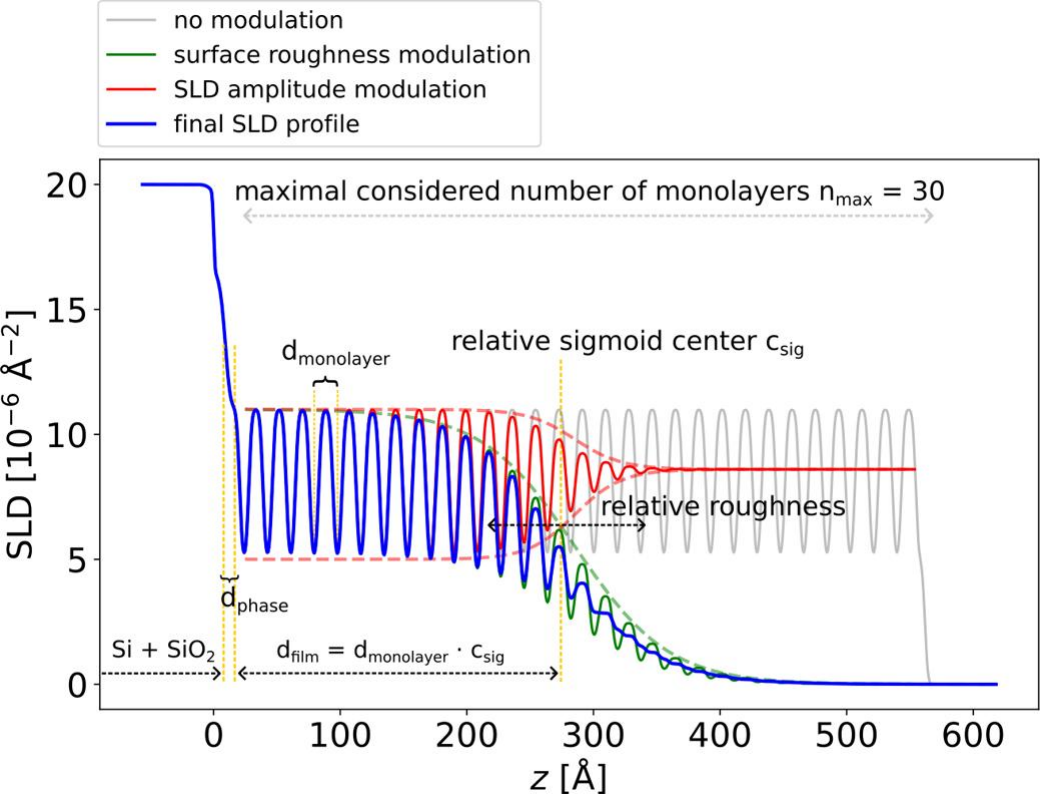
Physics-informed parameterization of the SLD profile for a thin film consisting of repeating identical monolayers on top of a substrate. The grey curve shows the base SLD profile of the monolayers, the green curve shows the SLD profile with surface roughness and the red curve shows the modulation of the SLD amplitude. The blue curve represents the final SLD profile.

In this subsection, instead of a box model parameterization, we employ a physics-informed parameterization of a complex multilayer structure, as illustrated in Fig. 10 ▸. The physical scenario is the following. On top of a silicon/silicon oxide substrate we consider a thin film composed of repeating identical monolayers (grey curve in Fig. 10 ▸), each monolayer consisting of two boxes with distinct SLDs. A sigmoid envelope modulating the SLD profile of the monolayers defines the film thickness and the roughness at the top interface (green curve in Fig. 10 ▸). A second sigmoid envelope can be used to modulate the amplitude of the monolayer SLDs as a function of the displacement from the position of the first sigmoid (red curve in Fig. 10 ▸). These two sigmoids allow one to model a thin film that is coherently ordered up to a certain coherent thickness but becomes incoherently ordered or amorphous towards the top of the film. Such a scenario is sometimes encountered when Kiessig and Laue fringes show different periods. In addition, a layer between the substrate and the multilayer is introduced to account for the interface structure, which does not necessarily have to be identical to the multilayer period. This ‘phase layer’ (*i.e.* a layer that strongly influences the relative scattering phase between the substrate and the multilayer) is important, as the relative phase between a strong substrate reflection and a multilayer Bragg reflection can lead to very different shapes of the curve around the Bragg reflection for constructive or destructive interference.

**Figure 11:**
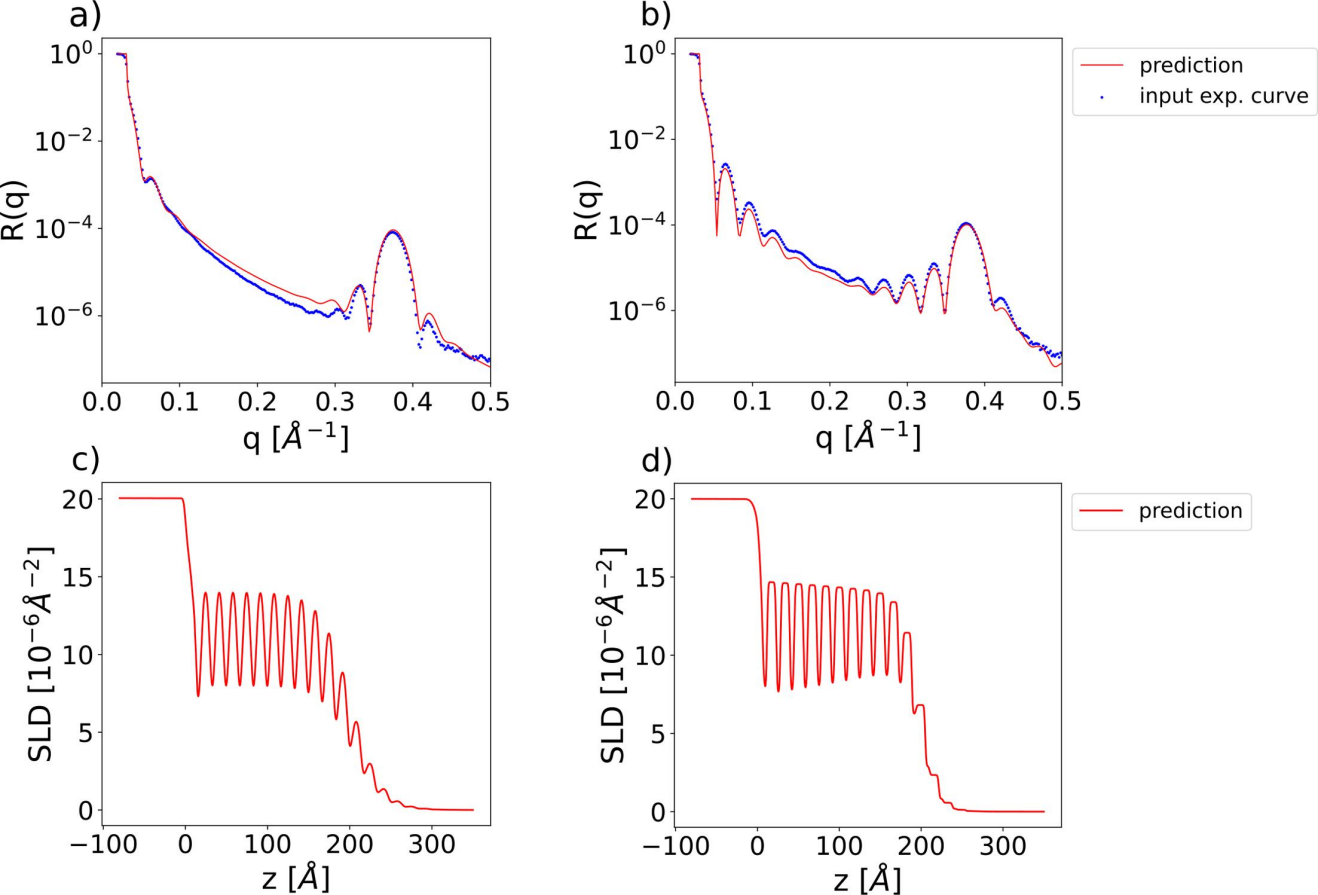
(*a*)–(*b*) Examples of input experimental reflectivity curves of DIP monolayers grown on top of a silicon/silicon oxide substrate (blue markers) with the corresponding neural network predictions (red lines) for the model with physics-informed parameterization. (*c*)–(*d*) Predicted SLD profiles corresponding to the reflectivity curves in the top row.

The 17 parameters describing the model together with their training ranges are displayed in Table 1 ▸. For experimental XRR curves of DIP monolayers, measured on a laboratory X-ray source, we use prior knowledge about this system (layer spacing, substrate SLDs, approximate SLD values for the two boxes in the monolayer) to set suitable prior bounds. Fig. 11 ▸ shows input experimental curves together with the neural network predictions for the model with physics-informed parameterization. Again the prediction quality is good, demonstrating the potential of the proposed method to fit experimental data in increasingly complex scenarios such as multilayer structures featuring Bragg peaks. Note that in this case the gradient descent polishing procedure introduced in previous studies (Greco *et al.*, 2022 ▸) can further improve the fit, and it can be performed both with the 17 introduced parameters and with the full set of box model parameters, allowing the final solution potentially to evolve beyond the designed parameterization.

### 4.4. Model with Fourier neural operator embedding network

**Figure 12:**
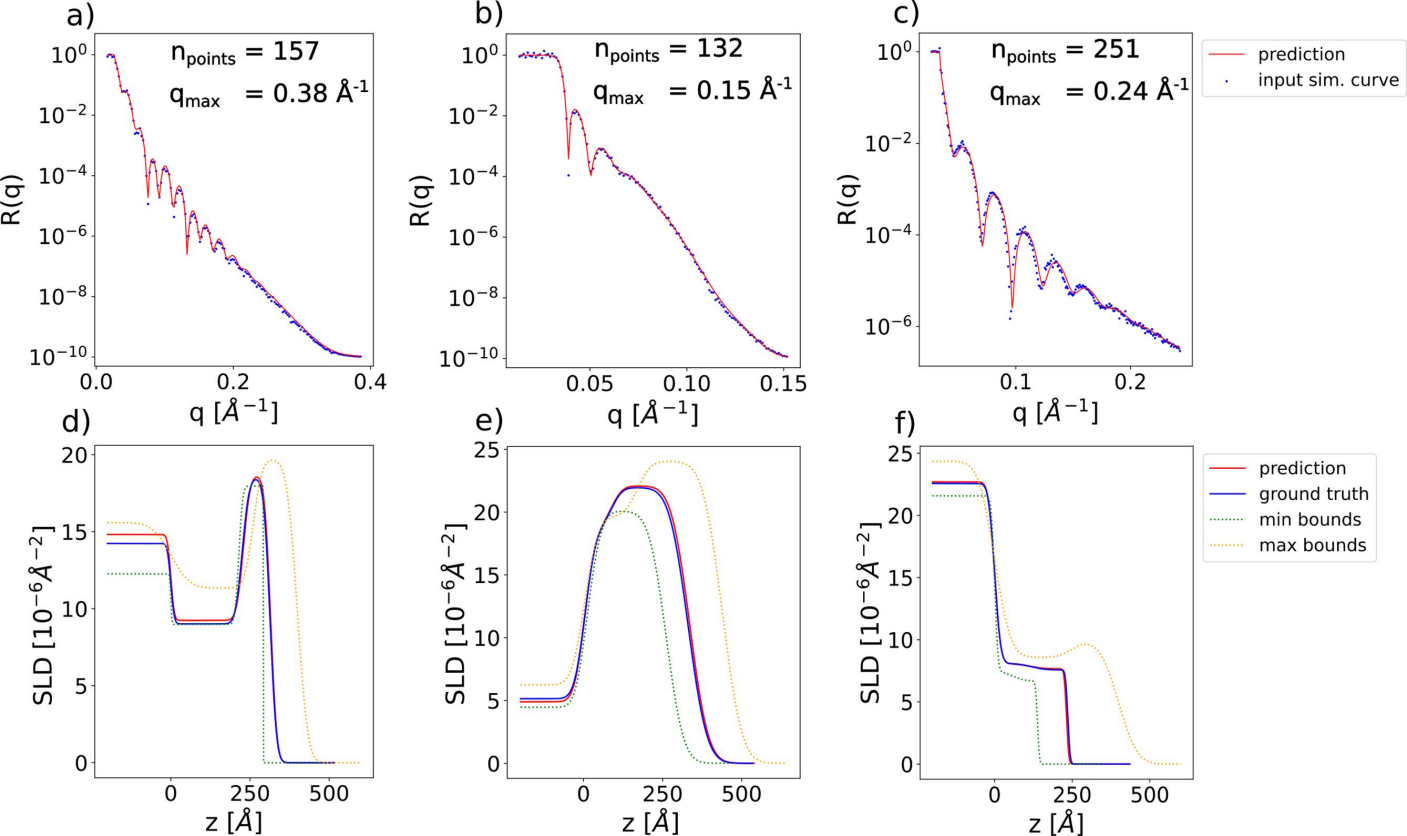
(*a*)–(*c*) Examples of input simulated reflectivity curves with variable discretizations (blue markers) and the corresponding neural network predictions (red lines) for a two-layer model with the FNO embedding network. (*d*)–(*f*) Ground-truth (blue) and predicted (red) SLD profiles corresponding to the reflectivity curves in the top row. SLD profiles corresponding to the minimum (green) and maximum (yellow) prior bounds used for the prediction are also shown as dotted lines. These minimum and maximum bound profiles should not be visually interpreted as an envelope encasing the ground-truth or predicted SLD profiles.

In this subsection, we show the results obtained when using the FNO as the embedding network instead of the 1D CNN used in the previous sections. The number of points in the simulated curves is in the range [128, 256], the minimum value of *q* is in the range [0.01, 0.03] Å^−1^ and the maximum value of *q* is in the range [0.15, 0.4] Å^−1^. We consider a two-layer box model with parameter ranges [0, 300] Å for the thicknesses, [0, 60] Å for the roughnesses and [0, 25] × 10^−6^ Å^−2^ for the SLDs. The ranges of the prior bound widths are [0.01, 300] Å for the thicknesses, [0.01, 60] Å for the roughnesses and [0.01, 4] × 10^−6^ Å^−2^ for the SLDs. Due to increased memory demands the batch size is reduced to 1024. Fig. 12 ▸ shows input curves with variable numbers of points and *q* ranges, together with the neural network predictions (top row), and the corresponding SLD profiles (bottom row). By using an FNO as the embedding network, our approach is successfully extended to curves with variable discretizations. A disadvantage in this scenario is that the network takes longer to converge.

## 5. Conclusions

In this study, we have addressed the ambiguity related to the phase problem as a primary obstacle in machine-learning-based approaches for extracting information from X-ray reflectivity and neutron reflectivity data. The ambiguity in the space of possible solutions increases the larger the considered parameter space becomes. This prevents the successful training of neural networks for complex multilayer structures with many free parameters. Therefore, previous solutions were limited to relatively simple layer structures with just a few parameters. To tackle this issue, we have proposed a procedure that enables training of a neural network on a continuous range of smaller subspaces of a large parameter space.

Our method allows users to incorporate prior experimental knowledge by specifying upper and lower bounds for each parameter during inference. This approach overcomes the limitations in existing machine learning methods, as it allows training of networks with a larger number of parameters and expanded parameter ranges, while still enabling proper convergence.

The proposed approach is a natural way of tackling the inverse problem in reflectometry as it resembles the standard workflow of conventional fitting techniques routinely used in the analysis of reflectometry data, *i.e.* choosing a proper parameterization of the SLD profile, setting prior bounds for the parameters and obtaining a single solution which best fits the data. We emphasize though that in some cases Bayesian analysis of the data can be preferred by researchers in order to understand the correlations between the parameters.

We have validated the effectiveness of our approach by training networks using different physical models: two-layer and five-layer box model parameterizations, and a specialized parameterization for repeating identical monolayers. In contrast to previous work, our approach scales favourably when increasing the complexity of the inverse problem, giving good predictions even for the challenging five-layer multilayer model.

We note that the proposed approach can be adapted to tackling other inverse problems in science affected by the non-uniqueness issue.

## Figures and Tables

**Table 1:** Parameters of the model with physics-informed parameterization of the SLD profile, together with the parameter ranges and the ranges of the prior bound widths used for training Some of the parameters are relative with respect to the monolayer thickness.

Parameter	Parameter range	Prior bound width range
Monolayer thickness	[10, 20] Å	[0.1, 10] Å
Relative roughness of the monolayer interfaces	[0, 0.3]	[0.1, 0.3]
SLD of the first box in the monolayer	[0, 20] × 10^−6^ Å^−2^	[0.1, 5] × 10^−6^ Å^−2^
SLD difference between the second and first boxes in the monolayer	[−10, 10] × 10^−6^ Å^−2^	[0.1, 5] × 10^−6^ Å^−2^
Fraction of the monolayer thickness belonging to the first box	[0.01, 0.99]	[0.01, 1]
Roughness of the silicon substrate	[0, 10] Å	[0.01, 10] Å
SLD of the silicon substrate	[19, 21] × 10^−6^ Å^−2^	[0.01, 2] × 10^−6^ Å^−2^
Thickness of the silicon oxide layer	[0, 10] Å	[0.01, 10] Å
Roughness of the silicon oxide layer	[0, 10] Å	[0.01, 10] Å
SLD of the silicon oxide layer	[17, 19] × 10^−6^ Å^−2^	[0.01, 2] × 10^−6^ Å^−2^
SLD of the phase layer	[0, 25] × 10^−6^ Å^−2^	[0.01, 25] × 10^−6^ Å^−2^
Relative thickness of the phase layer	[0, 1]	[0.01, 1]
Relative roughness of the phase layer	[0, 1]	[0.01, 1]
Relative position of the first sigmoid (total film thickness)	[0, 25]	[0.1, 25]
Relative width of the first sigmoid	[0, 5]	[0.1, 5]
Relative position of the second sigmoid (coherently ordered film thickness)	[−10, 10]	[0.1, 20]
Relative width of the second sigmoid	[0, 20]	[0.1, 20]
